# Prevalence and factors associated with nonalcoholic fatty liver disease in shanghai work-units

**DOI:** 10.1186/1471-230X-12-123

**Published:** 2012-09-14

**Authors:** Xiaona Hu, Yiqin Huang, Zhijun Bao, Yiqian Wang, Dongmei Shi, Fang Liu, Zhanjuan Gao, Xiaofeng Yu

**Affiliations:** 1 Department of Gastroenterology, Huadong Hospital, Fudan University, Shanghai, 200040, China; 2 Department of Internal Medicine, Shanghai Medical College, Fudan University, 221 West Yan An Road, Jing’an District, Shanghai, 200040, China

**Keywords:** Prevalence, Risk factors, Nonalcoholic fatty liver

## Abstract

**Backgrounds:**

Nonalcoholic fatty liver disease (NAFLD) has become the most common chronic liver disease in Asians. However, data on prevalence and factors associated with NAFLD in Asians are lacking. The aim of this study is to investigate the prevalence of NAFLD in Shanghai employees to assess the relationship between NAFLD and age, gender, metabolic risk factors in this studied population.

**Methods:**

We selected 7152 employees of Shanghai work-units. Each of them underwent detailed medical history-taking, physical examination, laboratory assessments and abdominal ultrasonography. The diagnosis of NAFLD was done according to established criteria. Receiver operating characteristics (ROC) curves were applied to detect areas under the ROC curves for each index. Nominal logistic regression analysis was used to estimate the odds ratio for risk factors of NAFLD.

**Results:**

About 38.17% employees had NAFLD, more in men than in women. The prevalence of NAFLD increased with increasing age. In both genders, the prevalence of metabolic factors was higher in the NAFLD group. Body max index, waist circumference, weight-to-height ratio, blood pressure, blood glucose, total cholesterol, triglyceride, low density lipoprotein, high density lipoprotein and uric acid were found to have a diagnostic value for NAFLD. Body max index is a better index for diagnosing NAFLD. Uric acid is a new diagnosing index not inferior to lipid metabolic factors. Metabolic factors can increase the risk of NAFLD up to 1.5 ~ 3.8 times.

**Conclusions:**

Older age, male gender, metabolic factors such as obesity, abdominal obesity, dyslipidemia, hypertension or type 2 diabetes are risk factors for NAFLD. Prevalence of NAFLD in Shanghai employees is high. Prevention is extremely important. Those achieve the critical point should have early intervention.

## Backgrounds

Nonalcoholic fatty liver disease (NAFLD) is a metabolic disorder characterized by excessive triglyceride accumulation in hepatocytes. Nowadays it has become a major public health hazard worldwide. Estimates of current prevalence rates range from 24% to 42% in Western countries and 5% to 40% in Asian countries [1-3]. In the USA, it is estimated that approximately a third of the general population has NAFLD [2,4]. In Japan, the prevalence is 9 ~ 30% [1,5,6]. And the prevalence of NAFLD in the general population of China varies from 5% to 24%. A combination of lifestyle, environmental, older age, gender, steroid hormone metabolism, genetic predisposition and metabolic factors play a role in the pathogenesis of NAFLD [7-10]. Genetic predisposition, overabundance of calorie-rich food and lack of physical activity contribute to development of obesity. Obesity is a pro-inflammatory state that leads to insulin resistance (IR), which is closely associated with NAFLD development and progression [7,11]. Advancing age decreases the hepatic metabolism of LDL cholesterol, increases abdominal adiposity and fat deposition in muscles, liver, and bone marrow, resulting in deleterious metabolic consequences of IR [8]. An ethnic variation in the distribution of NAFLD has also been suggested with Hispanics having the highest prevalence (45-58%), followed by Caucasians (33-44%) and African Americans (24-35%) [12,13].

NAFLD consists of a wide spectrum of conditions, ranging from simple steatosis to nonalcoholic steatohepatitis (NASH) which can progress to cirrhosis and hepatocellular carcinoma (HCC). It is reported that almost 10% ~ 20% of individuals with NAFLD have NASH, 10% ~ 15% of individuals with NASH progress to cirrhosis [14]. In patients with cirrhotic NASH, HCC and liver failure are the main causes of morbidity and mortality (5-year cumulative HCC development rate 11.3%, 5-year survival rate 75.2%, respectively) [15]. A “two-hit hypothesis” was proposed to explain the pathogenesis and progression of NAFLD. It was proposed that hepatocyte triglyceride accumulation resulting from metabolic imbalance (obesity, IR and diabetes) is the leading factor to steatosis (the “first hit”) and that the lipid-laden hepatocytes are then vulnerable to injurious processes (the “second hit”) such as cytokines and oxidative stress [7]. In this hypothesis, obesity and insulin resistance are the key pathogenic factors associated with NAFLD.

NAFLD has a multifactorial etiology and a combination of environmental, genetic and metabolic factors play a role in the development of advanced disease. So the pathophysiology of NAFLD is not fully understood. Therefore, study of the prevalence of NAFLD and identifying its risk factors would be critically important. China is a country with a large scale and many nations. Regional variations can be striking. Shanghai is an international city with more westernized lifestyle. The prevalence may be higher than the general population. Therefore, we conducted a study to determine the prevalence of and factors associated with NAFLD in Shanghai employees.

## Methods

### Subject recruitment

We assigned a number to each of 16 urban districts of Shanghai, and randomly selected three districts (Xuhui Districts, Jing’an Districts and Changning Districts). Of all the work-units we randomly selected three departments in each district. Then we performed medical check-ups at Huadong Hospital during 2011.

About 7152 adult participants, aged 18 ~ 65 years old, were enrolled in the study. The study was approved by the Research Ethics Committee of Huadong Health Bureau. The purpose of the study, procedures to be carried out, potential hazards and benefits were explained prior to obtaining an informed written consent. Each consenting adult underwent a detailed medical history-taking, physical examination, laboratory assessment and abdominal ultrasonography (US) carried out by hepatologists trained at the same institution to ensure interobserver consistency.

Information was gathered on sociodemographic variables, past history of liver disease, presence of co-morbidities, medical history and health-related habits such as smoking and drinking. Age, sex, occupation and education were gathered as sociodemographic variables. Past history covered previous or present diagnoses of hepatitis B or C infection, biliary diseases, surgical interventions and other chronic liver diseases. Presence of co-morbidities included hypertension, type 2 diabetes (T2DM) and obesity. Alcohol intake was assessed using two open questions: ‘How often did you have a drink containing alcohol per week in the past 6 months?’ and ‘How many glasses did you have on a typical day when you were drinking in the past 6 months?’ In the second question, one glass of alcoholic beverage was assumed to contain 10 g of alcohol. From these two questions, we calculated an average daily intake of alcohol.

In accordance with the guidelines, Subjects diagnosed with NAFLD had to fulfill the following criteria: no history of current or past excessive alcohol consumption, defined by an average daily consumption of alcohol intake > 20 g/day (140g/week) in males and > 10 g/day (70g/week) in females; no history of systemic illness known to cause fatty liver disease; not receiving or have recently received hepatotoxic drugs; negative tests for the presence of hepatitis B surface antigen and antibody to hepatitis C virus; absence of history and clinical, biochemical and US findings consistent with cirrhosis and other chronic(autoimmune, celiac disease, genetic disorders such as Wilson’s disease and alpha-1-antitrypsin deficiency) liver diseases; fulfilled the criteria with fatty liver under abdominal ultrasonography.

### Physical examination

Weight, standing height and waist circumference (WC) were measured in a standardized fashion by a trained examiner. WC measurement was made midway between the last rib and the iliac crest. The standing height and WC measurement were made at minimal inspiration to the nearest 0.1cm. Body mass index (BMI) was calculated as weight (kg) / stature (m^2^), and weight-to-Height ratio calculated as WC divided height. Readings of clinical blood pressure (BP) were obtained in the left arm of patients in the supine position, after 5 min of quiet rest, with a mercury sphygmomanometer. A minimum of three BP readings were taken on three separate occasions at least 2 weeks apart. Baseline BP values were the average of the last two of three consecutive measurements obtained at intervals of 3 min.

### Laboratory assessments

Antecubital venous blood samples were taken from all subjects after a 12h overnight fast. Using a multichannel autoanalyzer, we measured serum levels of alanine transarninase (ALT), total cholesterol (TC), triglyceride (TG), Low Density Lipoprotein cholesterol (LDL-C), High Density Lipoprotein cholesterol (HDL-C), fasting plasma glucose (FPG), creatinine (Cr) and uric acid (UA). Those participants with evidence of hepatic steatosis or abnormal blood tests of liver function had further investigations performed including serology of hepatitis B and C, ceruloplasmin, ferritin, alpha-1-antritrypsin level and phenotype and autoimmune markers such as antinuclear antibody (ANA), antismooth muscle antibody (SMA), and antimitochondrial antibody (AMA). Oral glucose tolerance test (OGTT) was performed on the subjects with abnormal fasting glucose except those with a previous diagnosis of diabetes.

### Ultrasonographic examination

Abdominal US was performed in all subjects by two hepatologists who were trained at the same institution and unaware of the clinical and laboratory data. Fatty liver was diagnosed in the presence of two of the three following criteria: increased hepatic echogenicity compared to the spleen or the kidneys, blurring of liver vasculature and deep attenuation of the ultrasonographic signal [16]. When the hepatic steatosis reaches 33%, the detection sensitivity is nearly 100%. So this has an adequate threshold for detection of steatosis when more than 33% of hepatocytes contain fat on liver histology [17,18].

### Definitions

According to current guidelines, patients with a systolic blood pressure (SBP) ≥ 140 mmHg and/or diastolic blood pressure (DBP) ≥ 90 mmHg were defined as hypertensive [19]. Causes of secondary hypertension were excluded by clinical and biochemical tests. Impaired fasting glucose and T2DM were defined by the American Diabetes Association criteria revised in 2003 [20]. Metabolic abnormalities were diagnosed following the Third Report of the National Cholesterol Education Expert Panel on Detection, Evaluation, and Treatment of High Blood Cholesterol in Adults, modified by the criteria of obesity proposed for Asians by the Steering Committee of the Regional Office for the Western Pacific Region of WHO (WPRO) [21,22]. The diagnosis of NAFLD was based on guidelines for the assessment and management of non-alcoholic fatty liver disease in the Asia-Pacific region [23,24].

### Statistical analyses

All statistical analyses were performed using SPSS 16.0. Data is expressed as the mean ± standard deviation or median [inter-quartile range (25%-75%)] or as percentage. Differences between groups were tested using an independent two-sample t-test or Maan-Whitney U-test for continuous variables, and the Pearson chi-square test was used to test for differences in the distribution of categorical variables. Nonparametric methods were carried out for non-normally distributed values. In each gender, receiver operating characteristics (ROC) curves were applied to detect the sensitivity, specificity and areas under the ROC curves (AUCs) for each index (normal vs abnormal) and NAFLD (absent vs present). Nominal logistic regression analysis was applied to analyze independent relationships of risk factors for NAFLD. All provided *P*-values represent the results of two-sided tests. *P*-values < 0.05 were considered statistically significant.

## Results

### General characteristics of participants

Overall, 7152 subjects enrolled in the study, 4330 subjects were men, while 2822 subjects were women. As shown in Table 1, the prevalence of NAFLD in general was 38.17% (47.88% for men and 23.28% for women). There is a significant difference in gender between NAFLD and non NAFLD group (51.04% for male and 48.96% for female in non NAFLD group,75.93% for male and 24.07% for female in NAFLD group, χ2 = 437.87, p < 0.0001). In males, the average age of the subjects with NAFLD was 46.82 ± 13.50, and 40.29 ± 14.76 in those without NAFLD. In females, the average age in NAFLD and non NAFLD group was 50.95 ± 11.58 and 38.28 ± 12.15 respectively. There were statistical differences in BMI, WC, weight-to-height ratio, SBP, DBP, TG, TC, LDL, HDL, UA, FPG and ALT between NAFLD group and non NAFLD group in both genders. There was no difference in Cr. Metabolic factors such as BMI, BP, TG, TC and FPG in NAFLD group were significantly higher than non NAFLD group.

**Table 1:** Baseline characteristics, biochemical tests and metabolic characteristics of subjects

	**Male (n=4330)**			**Female (n=2822)**		
	**Non NFALD(n=2257)**	**NALFD (n=2073)**	***P*-value**	**Non NALFD(n=2165)**	**NALFS (n=657)**	***P*-value**
Age (years)	40.29±14.76	46.82±13.50	<0.01	38.28±12.15	50.95±11.58	<0.01
BMI (kg/m^2^)	22.56±2.81	26.12±2.88	<0.01	21.24±2.56	25.34±2.88	<0.01
WC (cm)	78.69±9.79	89.69±11.45	<0.01	70.79±6.85	81.14±9.79	<0.01
W/H	0.46±0.06	0.52±0.06	<0.01	0.44±0.04	0.51±0.06	<0.01
SBP(mmHg)	117.42±14.64	127.99±17.08	<0.01	113.05±14.49	128.44±17.70	<0.01
DBP(mmHg)	71.99±12.03	80.45±12.43	<0.01	69.09±10.48	76.87±11.27	<0.01
TG(mmol/L)	1.0[0.8~1.5]	1.8[1.3~2.6]	<0.01	0.8[0.6~1.1]	1.5[1.1~2.1]	<0.01
TC(mmol/L)	4.40±0.81	4.89±0.89	<0.01	4.51±0.84	5019±1.00	<0.01
ALT(U/L)	19[14~25]	29[21~44]	<0.01	13[10~17]	21[16~30]	<0.01
UA(μmol/L)	353.00±69.93	385.16±76.64	<0.01	261.27±52.11	303.21±67.56	<0.01
FPG(mmol/L)	4.94±0.93	5.35±1.39	<0.01	4.83±0.71	5.37±1.34	<0.01
CR(μmol/L)	75.86±10.76	75.36±11.54	0.146	55.01±7.84	55.21±8.82	0.586
HDL(mmol/L)	1.78±0.42	1.43±0.36	<0.01	1.98±0.40	1.57±0.38	<0.01
LDL(mmol/L)	2.73±0.69	3.13±0.77	<0.01	2.80±0.71	3.31±0.85	<0.01

Values, mean±standard error of the mean (S.E.M).

BMI: body mass index; WC: waist circumference; W/H: weight to height ratio; SBP: systolic blood pressure; DBP: diastolic blood pressure; TG: Triglycerides; TC: total cholesterol; UA: Uric acid; FPG: Fasting plasma glucose; CR: Creatinine; HDL: High Density Lipoprotein cholesterol; LDL: Low Density Lipoprotein cholesterol.

### Age-specific prevalence of NAFLD

**Figure 1:**
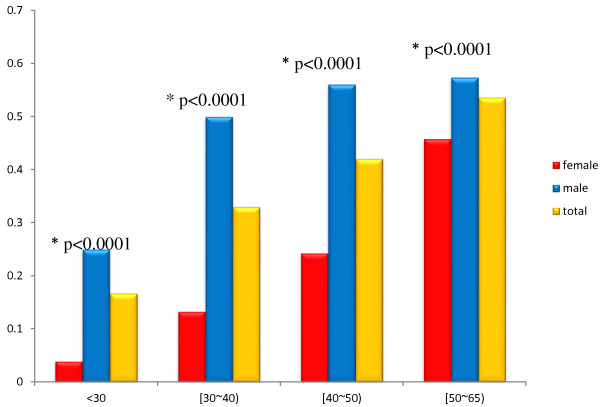
**Age-specific prevalence of NAFLD.** The prevalence of NAFLD increased according to age(trend chi-square value = 23.7292, p < 0.0001in total; chi-square value = 15.4859, p < 0.0001in male; trend chi-square value = 19.0515, p < 0.0001in female). The peak prevalence was in the 50-65 age group, up to 54% persons had NAFLD in total. The prevalence of NAFLD in males was significantly higher than females within the same age group (25% vs 3.89% in younger than 30- year old age group, 50% vs 13.29% in 30-39 age group, 56.06% vs 24.27% in 40-49 age group, and 57.35% vs 45.79% in 50-64 age group). In female, the prevalence increased dramatically after 50 years old. *The contraction between male and female in the same age group, *p* < 0.0001.

As showed in Figure 1, the prevalence of NAFLD increased according to age (trend chi-square value = 23.7292, p < 0.0001 in total; chi-square value = 15.4859, p < 0.0001 in male; trend chi-square value = 19.0515, p < 0.0001 in female).The peak prevalence was in the 50–65 age group, up to 54% of subjects had NAFLD in total. The prevalence of NAFLD in males was significantly higher than females within the same age group (*p* < 0.0001).

### Prevalence of metabolic syndrome

As shown in Table 2, the prevalence of metabolic factors in non NAFLD population was 8.19% for obesity, 7.61% for abdominal obesity, 11.18% for BP > 130/85mmHg or hypertension, 8.74% for dyslipidemia and 3.71% for FPG > 5.60mmol/L or T2DM. The prevalence of NAFLD combined with metabolic factors was 22.44% for obesity, 17.93% for abdominal obesity, 18.64% for BP > 130/85mmHg or hypertension, 19.85% for dyslipidemia and 7.69% for FPG > 5.60mmol/L or T2DM respectively.

**Table 2:** Prevalence of metabolic syndrome

		**Male**			**Female**			**Total**	
	**NAFLD (n(%))**	**Non NAFLD (n(%))**	**P**	**NAFLD (n(%))**	**Non NAFLD (n(%))**	**P**	**NAFLD (n(%))**	**Non NAFLD (n(%))**	**P**
obesity	1279(29.53)	419(9.68)	.000	326(11.55)	167(5.92)	.000	1605(22.44)	586(8.19)	.000
Abdominal obesity	966(22.31)	338(7.81)	.000	316(11.20)	206(7.30)	.000	1282(17.93)	544(7.61)	.000
BP>130/85 or HBP	1012(23.37)	498(11.50)	.000	321(11.37)	302(10.70)	.000	1333(18.64)	800(11.18)	.000
dyslipidemia	1137(26.26)	396(9.15)	.000	283(10.03)	229(8.11)	.000	1420(19.85)	625(8.74)	.000
FPG>5.6 or 2-DM	420(9.70)	182(4.20)	.000	130(4.61)	83(2.94)	.000	550(7.69)	265(371)	.000

Metobolic syndrome: (1) Obesty: Body mass index ≥25kg/m2; abdominal obesity: ≥90cm(male), ≥80cm(female); (2) Blood pressure (BP)≥130/85mmHg Or hypertension (HBP); (3) Dyslipidemia: Triglycerides (TG)≥1.7mmol/L, and/or High Density Lipoprotein cholesterol (HDL)≤1.03mmol/L, (male) <1.29mmol/L (female); (4) Fasting blood glucose (FPG)≥5.6 mmol/L Or type 2 diabetes (2-DM).

In males, the prevalence of metabolic factors in the NAFLD group was significantly higher than in non NAFLD group (obesity 29.53% vs 9.68%, abdominal obesity 22.31% vs 7.81%, BP > 130/85mmHg or hypertension 23.37% vs 11.50%, dyslipidemia 26.26% vs 9.15% and FBG > 5.60mmol/L or T2DM 9.70% vs 4.20% respectively). In females, the comparison of the prevalence of metabolic factors in the NAFLD group and non NAFLD group was 11.55% vs 5.92% for obesity, 11.20% vs 7.30% for abdominal obesity, 11.37% vs 10.70% for BP > 130/85mmHg or hypertension, 10.03% vs 8.11% for dyslipidemia and 4.61% vs 2.94% for FBG > 5.60mmol/L or T2DM.

The difference of the prevalence of metabolic factors in the males and females NAFLD group was 29.53% vs 11.55% for obesity, 22.31% vs 11.20% for abdominal obesity, 23.37% vs 11.37% for BP > 130/85mmHg or hypertension, 26.26% vs 10.03% for dyslipidemia and 9.70% vs 4.61% for FBG > 5.60mmol/L or T2DM.

### Receiver operating curve analyses of age, biochemical tests and metabolic characteristics

Table 3 shows the AUC values of the subjects with NAFLD. After analysis, we found that the AUC values for BMI was higher than other metabolic factors in both genders (p < 0.05). In males, there were no significant statistical differences in weight-to-height ratio and WC (u = 1.51, p = 0.066). The AUC values for TG, TC, HDL, LDL were higher than UA (p < 0.01). There were no significant statistical differences in FPG and UA. In females, there were also no significant statistical differences in weight-to-height ratio and WC (u = 1.33, p = 0.093). The AUC values for TG and HDL were higher than UA (p < 0.01), but there were no significant statistical differences in TC, LDL, FPG and DBP.

**Table 3:** The AUC values of metabolic risk factors for NAFLD

	**Male**				**Female**			
**Variable(s)**	**Area**^**†**^	**S.E**^**‡**^	**95%CI**^**§**^	***P*-value**	**Area**^**†**^	**S.E**^**‡**^	**95%CI**^**§**^	***P*-value**
BMI	.818	.006	0.806~0.831	.000	.867	.007	0.853~0.882	.000
WC	.776	.007	0.762~0.790	.000	.830	.009	0.813~0.847	.000
WC/Height	.791	.007	0.778~0.804	.000	.846	.008	0.829~0.862	.000
SBP	.688	.008	0.673~0.704	.000	.757	.011	0.736~0.778	.000
DBP	.690	.008	0.674~0.706	.000	.699	.012	0.676~0.722	.000
CR	.477	.009	0.460~0.494	.008	.503	.013	0.477~0.529	.820
UA	.628	.008	0.611~0.644	.000	.688	.012	0.665~0.711	.000
FPG	.628	.008	0.612~0.645	.000	.712	.012	0.689~0.735	.000
TG	.775	.007	0.761~0.789	.000	.808	.010	0.789~0.827	.000
TC	.665	.008	0.649~0.681	.000	.700	.011	0.677~0.722	.000
HDL	.735	.008	0.720~0.749	.000	.772	.010	0.752~0.792	.000
LDL	.656	.008	0.640~0.672	.000	.687	.012	0.663~0.710	.000
TG/HDL	.777	.007	0.763~0.791	.000	.810	.010	0.791~0.829	.000

†The area under the ROC curve; ‡Standard error; § 95% confidence interval.

BMI: body max index; WC: waist circumference; WC /Height: weight to height ratio; SBP: systolic blood pressure; DBP: diastolic blood pressure; CR: Creatinine; UA: Uric acid; FPG: Fasting blood glucose; TG: Triglycerides; TC: total cholesterol HDL: High Density Lipoprotein cholesterol; LDL: Low Density Lipoprotein cholesterol.

### The adjusted odds ratios of metabolic risk factors for NAFLD

Table 4 shows the risk factors for NAFLD after being adjusted for age. In males, NAFLD was more likely to occur in subjects with high TG levels (OR 3.809), those with obesity (OR 3.304), abdominal obesity (OR 1.960), high blood pressure (OR 1.949), high FPG (OR 1.502) and low HDL (OR 1.596). Hypertriglyceridemia is the strongest associated factor in males, while BMI is the strongest associated factor in females (OR 3.806), followed by Hypertriglyceridemia (OR 3.381), abdominal obesity (OR 2.846), high FPG (OR 2.458), high BP (OR 2.053), and low HDL (1.801).

**Table 4:** The adjusted odds ratios of metabolic risk factors for NAFLD

	**Male**				**Female**			
**Variable(s)**	**β**	**S.E**	**O.R**	***P*-value**	**β**	**S.E**	**O.R**	***P*-value**
BMI	1.195	.014	3.304	0.000	1.336	.023	3.806	.000
WC	.673	.014	1.960	.000	1.046	.022	2.846	.000
BP	.667	.011	1.949	.000	0.719	.017	2.053	.000
FPG	.407	.015	1.502	.000	0.899	.024	2.458	.000
TG	1.337	.012	3.809	.000	1.218	.020	3.381	.000
HDL	.468	.021	1.596	.000	0.588	.024	1.801	.000
normal	-6.239	.036	0.002	.000	-8.096	.048	0.000	.000

β: Beta coefficient; S.E: Standard error; OR: odds ratio; BMI: Body mass index, ≥25kg/m2; WC: waist circumference, ≥90cm(male), ≥80cm(female); BP: blood pressure, ≥130/85mmHg Or hypertension (HBP); FPG: Fasting blood glucose, ≥5.6 mmol/L Or type 2 diabetes (2-DM); TG: Triglycerides, ≥1.7mmol/L; HDL: High Density Lipoprotein, ≤1.03mmol/L, (male) <1.29mmol/L (female).

## Discussion

Several studies have shown that NAFLD is the main cause of chronic liver disease [25]. In China, the prevalence of NAFLD varied widely due to differences in occupation, age, gender, life-style and regions studied [26]. Previous studies had reported the prevalence of NAFLD in the general population of central China, Chengdu (Southwest China), Guangdong (South China) and Shanghai (East China) as 24.5%, 12.5%, 17% and 15% respectively [27-29]. Regional variations within China can be striking. Due to the difficulty of carrying out large scale population survey, the true prevalence of the general population in China is still absent. In this study, we investigated the prevalence of NAFLD in employees in Shanghai to evaluate the trends of NAFLD prevalence rates. Our study reveals that the present prevalence of NAFLD in Shanghai is 38.17%, much higher than the previous study. Our study also investigated the prevalence of the components of metabolic syndrome. The prevalence of obesity, hypertension, dyslipidemia and T2DM are 30.63%, 29.82%, 28.59% and 11.4% respectively. Therefore, the difference of the present and previous prevalence could be attributed to the increasing prevalence of obesity, hypertension, insulin resistance and T2DM.

Our study investigated the employees in an age range of 18–64 years old. The study confirmed that the prevalence of NAFLD increases with increasing age and the peak prevalence of NAFLD was between 50–65 years old. However, it is difficult to generalize our results to the very old as our subjects were mainly the ‘younger old’ with few being aged > 65 years. Further studies focusing specifically on the ‘old’ with NAFLD are needed.

Gender difference in the relationship between metabolic risk factors and NAFLD was another important finding in this study. A study conducted in a Korean population reported that the prevalence of NAFLD was 35% for men and 16% for women [30]. Our study revealed that the prevalence of NAFLD was estimated to be 47.88% for men and 23.28% for women, higher than Koreans. The data of higher prevalence of NAFLD among men compared to women was also supported from studies in USA, Japan and India [31-33]. This gender difference of prevalence maybe attributed to higher prevalence of metabolic syndrome in men [31]. The national difference in prevalence may be owing to ethnicity and lifestyle differences. In this study, the average age in the male NAFLD group was younger than the female group. In the same age group, the prevalence of NAFLD in men was higher than women. Logistic regression analysis of risk factors for NAFLD revealed that high TG level was the most relevant factor for NAFLD in men, while obesity was the most strongly associated factor for NAFLD in women (obesity defined by BMI ≥ 25 kg/m^2^). These age and gender differences may be due to differences in prevalence of obesity and life-style-related disease.

All these differences above can be explained by genetic predisposition. A genetic underpinning for NAFLD is suggested by a number of studies [10]. In 2008, Romeo et al. conducted the first genome wide association study and reported the strongest genetic signal for the presence of fatty liver (*PNPLA3*, patatin-like phospholipase domain containing 3; rs738409). It was reported that *PNPLA3* and four additional genetic variants had a modest role for lipid metabolism. The *PNPLA3* gene is responsible for the difference in prevalence of fatty liver disease between ethnic groups. It could also be responsible for a lower prevalence of steatosis in males [34]. Apart from the *PNPLA3* gene, many other genes can also influence the development of obesity and NAFLD via affecting lipid metabolism, cytokines, fibrosis mediators and oxidative stress [10,35,36]. Genetic factors shed a light in the identification of individuals at risk to develop NAFLD and its progression.

Many studies have proposed that the risk factors for NAFLD included a high fat diet, a sedentary lifestyle, insulin resistance, metabolic syndrome and its components (obesity, hypertension, dyslipidemia and T2DM) [2,26,37]. In our study, the ROC curves revealed that BMI, WC, weight-to-height ratio, BP, FPG, TC, TG, LDL, HDL and UA have diagnostic value for NAFLD. In western countries, visceral obesity had been shown to have a more important role in the pathogenensis of NAFLD than overall obesity. So WC has been a well-known surrogate marker of abdominal fat accumulation. But our studies revealed that BMI was superior to WC, and BMI is a better index for diagnosing NAFLD. Recently, many studies recommended that weight-to-height was an index not affected by height, it may be a good index for detecting NAFLD. But our study found that there was no significant statistical difference in the AUCs of weight-to-height ratio and WC, suggesting that waist-to-height ratio and WC has the same value for detecting NAFLD.

Recent studies have reported that serum uric acid levels were associated with NAFLD [38-41].The most significant finding of this study is that UA has a more diagnostic value for NAFLD. Our study found that there is no significant statistical difference in the AUCs of FPG and UA in males. UA is not inferior to FPG in diagnosing NAFLD. There are no significant statistical differences in the AUCs of UA and TC, LDL, BG, DBP in females. UA is not inferior to TC, LDL, BG and DBP in diagnosing NAFLD.

Our logistic regression analysis revealed that each component of metabolic syndrome was independently associated with NAFLD. Obesity, central obesity, BP > 130/85mmHg and/or hypertension, dyslipidemia, high uric acid and FPG > 5.6mmol/L and/or T2DM can increase the risk of NAFLD by 1.5 ~ 3.8 times. Previous studies had reported that obesity was the strongest associated factor after adjusting for age, gender and other metabolic factors [30,42,43]. But we found that obesity was the most relevant factor in females, which can increase the risk of NAFLD by 3.8 times, while it is hypertriglyceridemia in males, which can increase the risk of NAFLD by 3.8 times. In males, obesity is secondary to hypertriglyceridemia, with 3.3 times risk increase for NAFLD.

The present study does have some limitations. First, we tried our best to exclude subjects with a history of habitual alcohol consumption in this study. However, since the limit of excessive alcohol use probably needs to be adjusted according to the size of body and gender, the exact amount of alcohol consumed and its alcoholic content were difficult to assess. Second, although histopathological findings remain the gold standard for diagnosing NAFLD, they cannot be applied to a large-scale population. Abdominal US is non-invasive, easily undertaken and has a sensitivity ranging from 60% to 94% and a specificity from 84% to 95% in detecting the presence of fatty liver. If the hepatic steatosis reaches 33%, sensitivity approaches 100% [18]. So we used abdominal US to diagnose fatty liver. But when the infiltration is of less than 30% of the hepatic content, it is difficult to detect the presence of fatty liver. Finally, other confounders such as physical activity should also be considered. Further studies are required to adjust for these possibly distorted associations and to validate these findings to other populations.

## Conclusion

In conclusion, NAFLD is the main etiology of chronic liver disease. The prevalence of NAFLD is increasing these years. The prevalence in Shanghai is much higher than the previous study in 2005. Older age, male gender, metabolic factors such as obesity, abdominal obesity, dyslipidemia, hypertension or type 2diabetes can increase the risk of NAFLD with 1.5 ~ 3.8 times. The end-stage of NASH is cirrhosis, HCC, and/or liver failure. NAFLD is also a risk factor for death caused by cardiovascular disease. Future work needs to deepen our understanding of the pathogenesis and progression, especially genetic determinants of NAFLD for prevention. Improved methods for early detection of NAFLD, such as improving imaging techniques, finding novel plasma biomarkers are urgently needed. The medicines for the treatment of NAFLD should continue to be tested to determine their long-term safety and efficacy and new optimal management of NAFLD is also important.

## Abbreviations

NAFLD: Nonalcoholic fatty liver disease; NASH: Nonalcoholic steatohepatitis; HCC: hepatocellular carcinoma; BMI: Body mass index; WC: waist circumference; BP: Blood pressure; SBP: systolic blood pressure; DBP: diastolic blood pressure; TG: Triglyceride; TC: Total cholesterol; HDL-C: High Density Lipoprotein cholesterol; UA: Uric acid; Cr: Creatinine; FPG: Fasting plasma glucose; ALT: Alanine transarninase; AST: Aspartate aminotransferase; ANA: antinuclear antibody; AMA: antismooth muscle antibody; OGTT: Oral glucose tolerance test; US: ultrasonographic scan; T2DM: type 2 diabetes; ROC: Receiver operating characteristics; AUCs: areas under the ROC curves.

## Competing interests

The authors declare that they have no competing interests.

## Authors' contributions

Xiaona Hu undertook data collection, analyzed the interviews and wrote the manuscript and revised it upon the editor’s and reviewers’ guidance.Yiqin Huang collected data, transcribed and analyzed the interviews. Yiqian Wang and Dongmei Shi contributed to the study design and gave comments on the manuscript. Fang Liu, Zhanjuan Gao and Xiaofeng Yu undertook data collection and took part in the qualitative analysis of the interviews. Zhijun Bao conceived the initial idea, contributed to each stage of the study development, analysis, reporting, reviewed the analysis and interpretation of data, corrected the first draft, co-designed the contents and revised the manuscript. All authors have participated in the design of the study and have commented critically on the initial manuscript and have approved the final version of the manuscript.

## Funding

Natural Science Foundation of Shanghai (11ZR1411600).

## Pre-publication history

The pre-publication history for this paper can be accessed here:

http://www.biomedcentral.com/1471-230X/12/123/prepub
